# Biological action of melatonin on target receptors in breast cancer

**DOI:** 10.1590/1806-9282.20231260

**Published:** 2024-04-22

**Authors:** Paulo Celso Pardi, José Antonio Orellana Turri, Luiza Helena Costa Moreira Bayer, Gabriela Bezerra Nóbrega, José Roberto Filassi, Ricardo dos Santos Simões, Bruna Salani Mota, Isabel Cristina Espósito Sorpreso, Edmund Chada Baracat, José Maria Soares

**Affiliations:** 1 Universidade de São Paulo, Faculdade de Medicina, Hospital das Clínicas, Departamento de Obstetrícia e Ginecologia, Laboratório de Investigação Médica em Ginecologia Estrutural e Molecular (LIM-58), Disciplina de Ginecologia – São Paulo (SP), Brazil.; 2 Universidade de São Paulo, Faculdade de Medicina, Hospital das Clínicas, Departamento de Obstetrícia e Ginecologia, Setor de Mastologia, Disciplina de Ginecologia – São Paulo (SP), Brazil.

## INTRODUCTION

Melatonin is a hormone involved in the body's circadian rhythms, acting through receptors and distinct second messenger pathways to regulate the cell cycle, proliferation, survival, apoptosis, DNA repair, and tumor suppression^1^.

In vitro and in vivo studies showed that melatonin may prevent DNA damage and tumor growth and be related to the modulation and gene expression of estrogen, leading to a protective effect against breast cancer due to its antioxidant, immunomodulatory, and anticarcinogenic properties^1-6^.

High artificial exposure to light at night is related to an increased risk of breast cancer [relative risk (RR)=1.17, 95% confidence interval (CI): 1.11–1.23], and the risk of breast cancer was reduced by 14% after melatonin treatment (RR=0.86, 95%CI 0.78–0.95), with a linear dose–response trend (p=0.003)^7,8^.

Some studies suggest that melatonin's antioncogenic properties are due to its angiogenesis and apoptosis properties, which prevent tumor growth in breast cancer cells^2^. Another mechanism is the inhibition of human breast cancer growth by inhibiting tumor metabolism through phospho-activation of the receptor kinases Ak strain transforming (AKT), extracellular signal-regulated kinase (ERK1/2), and transcription factors^4,9^.

This review seeks to synthesize the available studies and evidence related to the influence of melatonin on breast cancer to better understand this hormone's role in the prevention, treatment, or control of this disease^9,10^.

## METHODS

It was a narrative review of melatonin receptors in breast cancer. The search was performed in the PubMed database between 2018 and 2023. The descriptors used were melatonin; N-acetyl-5-methoxytryptamine; breast neoplasms; breast cancer; mammary cancer receptors; estrogen; estrogen receptor (ER); ER-alpha; ER-beta; progesterone; progesterone receptor (PR); human epidermal growth factor receptor 2 (HER2); apoptosis; programmed cell death; antineoplastic agents; anticarcinogenic agents; and antioxidants.

## LITERATURE REVIEW

Melatonin is a molecule composed of three essential components: an aromatic indole ring, an acetamide side chain, and a methylene group^11^. Melatonin is crucial in regulating circadian rhythms and sleep-wake cycles. In the context of breast cancer, melatonin receptors have gained significant attention due to their potential role in modulating tumor development and progression^11-14^.

Melatonin affects target cells by binding to specific receptors, such as melatonergic receptors (MT1 and MT2 receptors), which are transmembrane G-protein-coupled proteins^15,16^. Activation of these receptors can lead to an intracellular signaling cascade, resulting in several biological effects^17-19^. Melatonin also acts in cell cycle regulation by regulating the progression of the cell through the phases of cell division, inhibiting cell cycle progression in cancer cells, and preventing uncontrolled cell growth^16,20^. Melatonin may affect the expression of hormone receptors, such as ER and PRs, essential in regulating cell growth and proliferation in breast cancer^21^. Melatonin increases antineoplastic immunity by reducing telomerase activity and inhibiting the fatty acid uptake and metabolic pathways of fat and the angiogenesis through vascular endothelial growth factor (VEGF) messenger ribonucleic acid (mRNA) in MCF-7 cells, and inhibits proliferation, invasion, and migration^22,23^.

### Epidermal growth factor and insulin-like growth factor 1 receptors

Melatonin can induce programmed cell death, or apoptosis, in cancer cells, reducing tumor size and proliferation by inhibiting the production of growth factors related to cancer development such as epidermal growth factor (EGF) and insulin-like growth factor 1 (IGF-1)^24,25^.

### Estrogen and progesterone receptors

Approximately 60–75% of breast cancers express estrogen receptor alpha (ERα). Melatonin affects the expression and activity of the estrogen receptors, which regulate breast cell growth and division. Melatonin has been shown to decrease the expression of ER-alpha and ER-beta and reduce estrogen binding to these receptors in vitro and in animal studies^26^.

Melatonin modulates estrogen signaling in estrogen synthesis by reducing the gonadotropin action, disrupting the activation of estradiol receptors on breast tumors, regulating the enzymes involved in the biosynthesis of estrogens in other tissues, resulting in reduced estrogen-dependent tumor growth, and potentially reducing the risk of hormone receptor-positive breast cancer^27^.

Progesterone is another crucial hormone in breast cell development and proliferation. Melatonin may interfere with the action of progesterone receptors (PR), reducing their expression and activity in breast cancer cells^26,28^.

### HER 2 receptors

Approximately 20–30% of breast tumors have HER2-positive receptors, a growth factor promoting cell division. Preclinical studies indicate that melatonin may affect HER2 expression by decreasing its activity and inhibiting tumor growth^29,30^.

### MT1 and MT2 melatonin receptors

MT1 receptors are widely expressed in various tissues, including the breast, while MT2 receptors are mainly found in the brain and retina. Both receptors have distinct but overlapping roles in regulating cellular processes and influencing breast cancer biology^31^.

MT1 is associated with the G-receptor protease family, and MT2 is related to the hydrolysis of phosphoinositide and calcium. Activation of MT1 receptors in breast cancer cells inhibits cell proliferation, induces cell cycle arrest, and promotes apoptosis. These actions are mediated by inhibiting specific signaling pathways involved in cell growth and survival, such as the PI3K/AKT and ERK/MAPK pathways. MT1 activation also helps suppress the formation of new blood vessels (angiogenesis) within tumors, thereby limiting their supply of nutrients^32^.

In estrogen receptor alpha (ERα)-positive human breast cancer, melatonin, via the MT1 receptor, suppresses ERα mRNA expression and ERα transcriptional activity. Some studies suggest that MT2 activation may also increase the invasiveness and metastasis of breast cancer cells. This conflicting role of MT2 receptors in breast cancer requires further investigation to understand their underlying mechanisms^32-34^.

### Synergic effect of melatonin with anticancer chemotherapy and radiotherapy

The role of statins in combination with melatonin has been extensively investigated regarding its risk reduction in specific cancer types. Pravastatin, a statin medication, is a widespread chemotherapy used to treat high blood cholesterol levels and prevent heart attacks and strokes. The chemopreventive effects of pravastatin in combination with melatonin in a breast cancer experimental model were evaluated. Pravastatin alone suppressed tumor frequency by 20.5% and average tumor volume by 15% compared with controls. The combined administration of the drugs decreased tumor frequency by 69%^35^.

Doxorubicin is one of the most common chemotherapy drugs used to control breast cancer. Chemotherapeutic resistance, particularly to doxorubicin, represents a significant impediment to successfully treating breast cancer and is linked to elevated tumor metabolism, tumor overexpression, and/or activation of various families of receptor- and non-receptor-associated tyrosine kinases. Doxorubicin and other chemotherapy drugs are frequently employed as the initial treatment for individuals dealing with metastatic breast cancer or endocrine resistance. Similar to many other chemotherapy agents, doxorubicin often prompts resistance among patients. The capacity of melatonin to hinder the activation and expression of these kinases adds substantial backing to the emerging concept of melatonin functioning as a circadian-regulated kinase inhibitor^36^.

The synergistic use of doxorubicin and melatonin holds promise as a prospective approach to treating breast cancer, demonstrating combined antitumor and anti-apoptotic influences while regulating calcium influx and its associated ion channel receptors. The results suggest that melatonin not only enhances the actions of doxorubicin through the activation of calcium ion channel receptors and the promotion of apoptosis but also triggers the demise of breast cancer cells^36^.

Likewise, the emergence of resistance to tamoxifen and alternative endocrine treatments has emerged as a significant obstacle within endocrine therapy. This issue is pronounced, with an estimated 30–50% of patients harboring estrogen receptor-positive breast tumors exhibiting inherent resistance. Furthermore, while most patients initially respond, the eventual development of acquired resistance to tamoxifen is nearly universal^6^.

Augmenting cancer cells’ susceptibility to radiation is a paramount objective within clinical radiobiology. The oncostatic effects of melatonin hold particular significance for estrogen-dependent mammary tumors. The impact of co-administering ionizing radiation and melatonin on proteins engaged in estrogen biosynthesis within breast cancer cells has undergone thorough scrutiny. Preliminary treatment with melatonin prior to radiation significantly diminishes the presence of active estrogens at the cancer cell level, reducing the activity and expression of proteins integral to estrogen synthesis by 50%. Furthermore, melatonin elicits a twofold alteration in p53 expression in comparison to radiation treatment alone^22^.

## DISCUSSION

It is crucial to recognize that the impact of melatonin on individual receptor types can exhibit variations contingent on factors such as the cancer stage, concurrent genetic modifications, and patient-specific elements. There is a substantial amount yet to be unveiled regarding the interplay between melatonin and receptors in breast cancer. Consequently, further research is imperative to unravel these mechanisms and their complexities^27,32,34^.

Given that night-time melatonin significantly suppresses tumor kinase signaling, one could consider melatonin a broadly based "circadian-regulated broad kinase inhibitor" that exhibits potent antimetabolic, antiproliferative, and progressive/metastatic activity in breast cancer^12,37^.

Breast cancer patients could potentially encounter varying levels of nocturnal light exposure due to factors such as stress, sleep deprivation, or extended night shift work, resulting in disruptions to their circadian rhythms and a decrease in melatonin production^9^. This factor could contribute to inherent and potentially acquired resistance to multiple chemotherapy agents. Consequently, there arises the potential for a novel approach involving the administration of chemotherapy in a manner optimized for circadian rhythms, in conjunction with supplemental melatonin therapy, for breast cancer patients. It is a possibility that needs further studies to prove it.

## CONCLUSION

Melatonin action may act on cancer development and may reduce the risk of breast cancer. However, further studies are necessary.

## References

[1] Ahmad SB, Ali A, Bilal M, Rashid SM, Wani AB, Bhat RR (2023). Melatonin and health: insights of melatonin action, biological functions, and associated disorders. Cell Mol Neurobiol.

[2] Bhattacharya S, Patel KK, Dehari D, Agrawal AK, Singh S (2019). Melatonin and its ubiquitous anticancer effects. Mol Cell Biochem.

[3] Amin N, Shafabakhsh R, Reiter RJ, Asemi Z (2019). Melatonin is an appropriate candidate for breast cancer treatment: based on known molecular mechanisms. J Cell Biochem.

[4] Shim S, Park KM, Chung YJ, Kim MR (2021). Updates on therapeutic alternatives for genitourinary syndrome of menopause: hormonal and non-hormonal managements. J Menopausal Med.

[5] Veiga ECA, Simões R, Valenti VE, Cipolla-Neto J, Abreu LC, Barros EPM (2019). Repercussions of melatonin on the risk of breast cancer: a systematic review and meta-analysis. Rev Assoc Med Bras (1992).

[6] Dauchy RT, Xiang S, Mao L, Brimer S, Wren MA, Yuan L (2014). Circadian and melatonin disruption by exposure to light at night drives intrinsic resistance to tamoxifen therapy in breast cancer. Cancer Res.

[7] Yang WS, Deng Q, Fan WY, Wang WY, Wang X (2014). Light exposure at night, sleep duration, melatonin, and breast cancer: a dose-response analysis of observational studies. Eur J Cancer Prev.

[8] Schernhammer ES, Hankinson SE (2009). Urinary melatonin levels and postmenopausal breast cancer risk in the nurses’ health study cohort. Cancer Epidemiol Biomarkers Prev.

[9] Castro TB, Mota AL, Bordin-Junior NA, Neto DS, Zuccari DAPC (2018). Immunohistochemical expression of melatonin receptor MT1 and glucose transporter GLUT1 in human breast cancer. Anticancer Agents Med Chem.

[10] Gurunathan S, Qasim M, Kang MH, Kim JH (2021). Role and therapeutic potential of melatonin in various type of cancers. Onco Targets Ther.

[11] Fang L, Li Y, Wang S, Yu Y, Li Y, Guo Y (2019). Melatonin induces progesterone production in human granulosa-lutein cells through upregulation of StAR expression. Aging (Albany NY).

[12] Kong X, Gao R, Wang Z, Wang X, Fang Y, Gao J (2020). Melatonin: a potential therapeutic option for breast cancer. Trends Endocrinol Metab.

[13] Nasrabadi NN, Sargazi F, Shokrzadeh M, Abediankenari S, Hoseini SV, Najafi M (2018). Expression of MT1 receptor in patients with gastric adenocarcinoma and its relationship with clinicopathological features. Neuro Endocrinol Lett.

[14] Rohilla S, Singh M, Priya S, Almalki WH, Haniffa SM, Subramaniyan V (2023). Exploring the mechanical perspective of a new anti-tumor agent: melatonin. J Environ Pathol Toxicol Oncol.

[15] Hasan M, Marzouk MA, Adhikari S, Wright TD, Miller BP, Matossian MD (2019). Pharmacological, mechanistic, and pharmacokinetic assessment of novel melatonin-tamoxifen drug conjugates as breast cancer drugs. Mol Pharmacol.

[16] Hasan M, Browne E, Guarinoni L, Darveau T, Hilton K, Witt-Enderby PA (2020). Novel melatonin, estrogen, and progesterone hormone therapy demonstrates anti-cancer actions in MCF-7 and MDA-MB-231 breast cancer cells. Breast Cancer (Auckl).

[17] Lin PH, Tung YT, Chen HY, Chiang YF, Hong HC, Huang KC (2020). Melatonin activates cell death programs for the suppression of uterine leiomyoma cell proliferation. J Pineal Res.

[18] Li Y, Li S, Zhou Y, Meng X, Zhang JJ, Xu DP (2017). Melatonin for the prevention and treatment of cancer. Oncotarget.

[19] Panchenko AV, Tyndyk ML, Maydin MA, Baldueva IA, Artemyeva AS, Kruglov SS (2020). Melatonin administered before or after a cytotoxic drug increases mammary cancer stabilization rates in HER2/Neu mice. Chemotherapy.

[20] Park HK, Ryu MH, Hwang DS, Kim GC, Jang MA, Kim UK (2022). Effects of melatonin receptor expression on prognosis and survival in oral squamous cell carcinoma patients. Int J Oral Maxillofac Surg.

[21] Sang X, Li L, Rui C, Liu Y, Liu Z, Tao Z (2021). Induction of EnR stress by Melatonin enhances the cytotoxic effect of lapatinib in HER2-positive breast cancer. Cancer Lett.

[22] González-González A, González A, Rueda N, Alonso-González C, Menéndez JM, Martínez-Campa C (2020). Usefulness of melatonin as complementary to chemotherapeutic agents at different stages of the angiogenic process. Sci Rep.

[23] Ferreira CS, Carvalho KC, Maganhin CC, Paiotti AP, Oshima CT, Simões MJ (2016). Does melatonin influence the apoptosis in rat uterus of animals exposed to continuous light?. Apoptosis.

[24] Stauch B, Johansson LC, Cherezov V (2020). Structural insights into melatonin receptors. FEBS J.

[25] Talib WH, Alsayed AR, Abuawad A, Daoud S, Mahmod AI (2021). Melatonin in cancer treatment: current knowledge and future opportunities. Molecules.

[26] Targhazeh N, Reiter RJ, Rahimi M, Qujeq D, Yousefi T, Shahavi MH (2022). Oncostatic activities of melatonin: roles in cell cycle, apoptosis, and autophagy [Biochimie 200 (2022) 44-59]. Biochimie.

[27] Talib WH, Saleh S (2015). Propionibacterium acnes augments antitumor, anti-angiogenesis and immunomodulatory effects of melatonin on breast cancer implanted in mice. PLoS One.

[28] Viswanathan AN, Schernhammer ES (2009). Circulating melatonin and the risk of breast and endometrial cancer in women. Cancer Lett.

[29] Wang Q, Lu Q, Guo Q, Teng M, Gong Q, Li X (2022). Structural basis of the ligand binding and signaling mechanism of melatonin receptors. Nat Commun.

[30] Garaulet M, Lopez-Minguez J, Dashti HS, Vetter C, Hernández-Martínez AM, Pérez-Ayala M (2022). Interplay of dinner timing and MTNR1B type 2 diabetes risk variant on glucose tolerance and insulin secretion: a randomized crossover trial. Diabetes Care.

[31] Reiter RJ, Rosales-Corral SA, Tan DX, Acuna-Castroviejo D, Qin L, Yang SF (2017). Melatonin, a full service anti-cancer agent: inhibition of initiation, progression and metastasis. Int J Mol Sci.

[32] Samanta S (2022). Melatonin: a potential antineoplastic agent in breast cancer. J Environ Pathol Toxicol Oncol.

[33] Ghosh H, Rai S, Manzar MD, Pandi-Perumal SR, Brown GM, Reiter RJ (2022). Differential expression and interaction of melatonin and thyroid hormone receptors with estrogen receptor α improve ovarian functions in letrozole-induced rat polycystic ovary syndrome. Life Sci.

[34] Wu J, Tan X (2022). The role of MTNR1B polymorphism on circadian rhythm-related cancer: a UK biobank cohort study. Int J Cancer.

[35] Orendáš P, Kubatka P, Bojková B, Kassayová M, Kajo K, Výbohová D (2014). Melatonin potentiates the anti-tumour effect of pravastatin in rat mammary gland carcinoma model. Int J Exp Pathol.

[36] Xiang S, Dauchy RT, Hauch A, Mao L, Yuan L, Wren MA (2015). Doxorubicin resistance in breast cancer is driven by light at night-induced disruption of the circadian melatonin signal. J Pineal Res.

[37] Goyal R, Gupta T, Bal A, Sahni D, Singh G (2020). Role of melatonin in breast carcinoma: correlation of expression patterns of melatonin-1 receptor with estrogen, progesterone, and HER2 receptors. Appl Immunohistochem Mol Morphol.

